# Real-world impact of the introduction of chemo-immunotherapy in extended small cell lung cancer: a multicentric analysis

**DOI:** 10.3389/fimmu.2024.1353889

**Published:** 2024-01-22

**Authors:** Laura Bonanno, Lorenzo Calvetti, Alessandro Dal Maso, Alberto Pavan, Loc Carlo Bao, Mattia De Nuzzo, Stefano Frega, Giulia Sartori, Alessandra Ferro, Giulia Pasello, Paolo Morandi, Giuseppe Aprile, Valentina Guarneri

**Affiliations:** ^1^ Medical Oncology 2, Veneto Institute of Oncology IOV - IRCCS, Padova, Italy; ^2^ Department of Oncology, Azienda ULSS 8 Berica, San Bortolo General Hospital, Vicenza, Italy; ^3^ Medical Oncology Department, Azienda ULSS 3 Serenissima, Dell’Angelo General Hospital, Mestre and SS Giovanni e Paolo General Hospital, Venezia, Italy; ^4^ Department of Surgery, Oncology and Gastroenterology, University of Padova, Padova, Italy

**Keywords:** small cell lung cancer, immune-checkpoint inhibitors, frail population, long-term clinical benefit, immune-related toxicity

## Abstract

**Background:**

Recent clinical trials demonstrated longer survival in extended small cell lung cancer (SCLC) patients treated with immunotherapy in addition to chemotherapy. However, the magnitude of benefit is modest and the impact in real-world setting has to be fully established.

**Methods:**

We collected clinical data and radiological imaging of patients affected by extended or relapsing SCLC and consecutively treated according to clinical practice between 2016 and 2023. As primary end-point, we compared pre-defined outcome indicators before and after the introduction of chemo-immunotherapy (May 2020): 6-month and 12-month progression free survival (PFS) rate, 12-month and 18-month overall survival (OS). Among those who were treated after May 2020, patients who did not receive immunotherapy according to treating physician’s choice were included in the analysis to minimize clinical selection bias.

**Results:**

The analysis included 214 patients: 132 (61.7%) were treated in an Academic cancer center and 82 (38.3%) in two community hospitals; 104 were treated before May 2020. Median PFS of the overall study population was 4.8 months (95% confidence interval [95% CI]: 4.4-5.4), median OS was 7.1 months (95% CI: 6.3-7.7). Estimated PFS and OS were significantly longer in patients treated after May 2020 with hazard ratio (HR) for PFS and OS of 0.61 (95% CI: 0.46-0.81, p < 0.001) and 0.70 (95% CI: 0.52-0.93, p = 0.015), respectively. 6-month PFS rate increased from 27% to 40% (p = 0.04) while 12-months PFS raised from 1% to 11% (p = 0.003). 12-month and 18-month OS rate increased from 15% to 28% (p = 0.03) and from 2.1% to 12% (p = 0.009), respectively. After May 2020 the median number of hospitalization days per patient decreased significantly and the incidence of severe AEs was similar. Among patients treated with chemo-immunotherapy, the onset of immune-related AEs was associated with improved PFS and OS (HR 0.55, 95% CI: 0.35-0.89, p = 0.012 and HR 0.47, 95%CI 0.28-0.77, p = 0.002, respectively).

**Conclusions:**

The real-world analysis shows a meaningful improvement of outcome indicators after the introduction of chemo-immunotherapy, with reduction of the duration of hospitalization, thus supporting the use of chemo-immunotherapy and the need for further biomarker research.

## 1 Introduction

Small-cell lung cancer (SCLC) accounts for about 15% of all new diagnoses of lung cancer. It is characterized by high biological aggressiveness and tendency to early metastatic spread (1, 2). About two thirds of patients are diagnosed with metastatic disease and their prognosis is very poor, with historically reported two-year overall survival (OS) rate inferior to 5% (3, 4). Standard systemic first-line treatment has been platinum-etoposide association for over two decades (5–7). The treatment was often characterized by rapid and deep responses but lead to median OS of approximately 10 months (8, 9). This treatment paradigm has recently changed with the introduction of combination strategies including platinum-etoposide and immune checkpoint inhibitors (ICIs) (2). Five randomized clinical trials have investigated the role of chemo-immunotherapy in extended SCLC (10–14) and the new standards of care refer to two pivotal trials assessing the superiority of platinum- etoposide plus atezolizumab (IMpower133) or durvalumab (CASPIAN) over standard chemotherapy alone (10, 11). Both studies showed a significant improvement in OS reaching median OS of 12.3 and 12.9 months for the experimental arm, respectively (10, 15). Although the benefit of the addition of immunotherapy is overall consistent along clinical trials, the modest entity of clinical benefit underlines the complexity of tumor biology and the need for predictive biomarkers and new combination strategies (16–20).

In addition, clinical trials included clinically selected population, while real-world (RW) patients affected by SCLC often have relevant comorbidities, older age and high symptoms burden. In this context, the fraction of RW patients complying with pivotal clinical trials inclusion/exclusion criteria is rather limited and previously estimated as about 36% in a retrospective cohort analysis in Caucasian population (21).

The aim of our study is to observe the clinical impact of the introduction of chemo-immunotherapy in RW patients with extended SCLC, referring to both Academic and Community general hospitals.

## 2 Methods

### 2.1 Patients

The study is a multicenter RW retrospective observational study analyzing a cohort of patients affected by extended SCLC and referring to three Oncology Departments belonging to the Italian Veneto region Oncology network, established by Regional government in 2013 (Rete Oncologica Veneta, ROV). The three institutions included one Academic center (Istituto Oncologico Veneto IOV – IRCCS, Padova) and two Community general hospitals (San Bortolo General Hospital, AULSS 8, Vicenza and Dell’Angelo General Hospital, AULSS 3, Mestre, Venezia).

We enrolled patients diagnosed with extended SCLC, from 2016 to 2023. Patients were staged according to the American Joint Committee of Cancer (22, 23). Stage III patients were included when radical-intent radiotherapy was not feasible. Other key inclusion criteria were feasibility of platinum-based chemotherapy, according to treating physician evaluation (at least one cycle of carboplatin/cisplatin plus etoposide performed) and availability of adequate follow-up. Medical history, radiological evaluations and details about treatment toxicity and clinical follow-up were collected from electronic medical reports and radiological imaging blinded review was performed. Patients were all treated according to clinical practice. Starting from May 2020 chemo-immunotherapy with carboplatin, etoposide and atezolizumab was approved in Italy (Italian Medicine Agency) following the results of IMpower133 study (10). No patients included were treated with durvalumab, since the drug was approved in Italy only after November 2022.

This study was approved by local ethics committees and conducted according to Helsinki declaration.

### 2.2 Study design and statistical analysis

Primary endpoint was to compare outcomes of patients diagnosed with extended SCLC before and after the introduction of chemo-immunotherapy, according to pre-defined clinical indicators. Primary clinical indicators included: 6-month and 12-month progression-free survival (PFS) rate, 12-month and 18-month OS rate. To limit potential selection bias related to clinical selection of patients for chemo-immunotherapy, all the patients were included in primary analysis, even those who did not receive immunotherapy after May 2020.

Secondary end-points were the rate of patients actually receiving chemo-immunotherapy in RW setting, the reasons for excluding them from immunotherapy treatment and hospitalization rate and per patient hospitalization duration, before and after the introduction of chemo-immunotherapy.

Clinical variables recorded included gender, smoke, age at diagnosis, baseline ECOG performance status (PS), brain, bone and liver metastases at baseline, and administered treatments.

Baseline complete blood count and lactate dehydrogenase (LDH) levels were recorded up to 14 days before starting first line systemic treatment. Neutrophils to lymphocytes ratio (NLR), derived neutrophils to lymphocytes ratio (dNLR, defined as neutrophils/[white blood cells count – neutrophils ratio]), platelet to lymphocytes ratio (PLR), lymphocytes to monocytes ratio (LMR) and lung immune prognostic index (LIPI) were calculated (24).

PFS was calculated from the date of initiation of systemic treatment until radiological progression of disease or death from any cause; OS was defined as the time from the initiation of treatment to death from any reason. Radiological response was evaluated according to the RECIST criteria v.1.1 (Response Evaluation Criteria in Solid Tumors) (25). The overall response rate (ORR) consisted of the proportion of patients obtaining partial response (PR) or complete response (CR) following treatment. The disease control rate (DCR) referred to the number of patients obtaining partial response, complete response or disease stability. Adverse events were reported according to the Common Terminology Criteria for Adverse Events (CTCAE), version 5.0. Categorical variables were summarized reporting proportions, while continuous variables were summarized with median and interquartile range (IQR). For correlation analysis, the Pearson’s chi-squared, Fisher’s exact test or Wilcoxon rank sum test were used, as appropriate. Kaplan-Meier curves were built for survival analysis and median PFS (mPFS) and median OS (mOS) along with 95% confidence interval (95% CI) were estimated, as well as survival rates at fixed time points with 95% CI. The log-rank test was used to compare survival curves, whereas cloglog fixed-point test was applied to compare survival rates between groups at fixed time points (26). Univariate and multivariate hazard ratios (HRs) and their 95% CI were estimated through Cox regression analysis. Statistical analysis was performed with R software (version 4.3.0, The R Foundation, Vienna, Austria).

## 3 Results

### 3.1 Study population and treatment

During the study period, 214 extended SCLC patients were included: 132 (61.7%) were treated in the Academic cancer center and 82 (38.3%) were treated in the two Community general hospitals (**Supplementary Figure 1**). One hundred and four patients underwent systemic treatment before chemo-immunotherapy while 110 were treated after chemo-immunotherapy introduction (**Supplementary Figure 1**).

**Table 1** depicts main clinical features both of the overall study population and of the two cohort of extended SCLC patient (before and after the introduction of chemo-immunotherapy). Overall, patients were mainly male, former or current smoker, median age at diagnosis was 70 years and 34% of patients were diagnosed with ECOG PS > 1. Clinical features, as well as potential prognostic and predictive factors appeared to be well-balanced between the two groups, in particular, the rate of ECOG PS > 1 patients, and brain and liver involvement were similar (**Table 1**).

**Table 1:** Clinical features of the study population.

Characteristic	Overall N = 214^1^	After CTIO introduction N = 110^1^	Before CTIO introduction N = 104^1^	p-value^2^
Sex				0.6
Male	135 (63.1%)	67 (60.9%)	68 (65.4%)	
Female	79 (36.9%)	43 (39.1%)	36 (34.6%)	
Smoke				>0.9
Actual	133 (63.6%)	71 (64.5%)	62 (62.6%)	
Former	74 (35.4%)	38 (34.5%)	36 (36.4%)	
Never	2 (1.0%)	1 (0.9%)	1 (1.0%)	
Previous limited disease	14 (6.5%)	11 (10.0%)	3 (2.9%)	0.051
Age at diagnosis	70 (64, 75)	69 (61, 75)	70 (64, 75)	0.5
Baseline ECOG PS				0.9
0-1	142 (66.4%)	72 (65.5%)	70 (67.3%)	
> 1	72 (33.6%)	38 (34.5%)	34 (32.7%)	
Brain metastases at baseline	46 (21.5%)	25 (22.7%)	21 (20.2%)	0.7
Bone metastases at baseline	56 (26.2%)	29 (26.4%)	27 (26.0%)	>0.9
Liver metastases at baseline	100 (46.7%)	51 (46.4%)	49 (47.1%)	>0.9

^1^ n (%); Median (IQR).

^2^ Fisher’s exact test; Wilcoxon rank sum test.

When we considered potential factors affecting outcome and response to treatment, we noticed that about 50% of patients received steroids at the start of first-line treatment (55.5% after the introduction of chemo-immunotherapy and 51.5% before the introduction of chemo-immunotherapy, p = 0.6), but the median daily dosage was significantly reduced after the introduction of chemo-immunotherapy (median prednisone-equivalent daily dosage: 13 mg after chemo-immunotherapy introduction *versus* 25 mg daily before chemo-immunotherapy introduction, p = 0.009).

Overall, first line treatment was carboplatin and etoposide in ninety patients (42%), cisplatin and etoposide in 35 patients (16.4%) and carboplatin, etoposide plus atezolizumab in 89 patients (41.6%) (**Table 2**).

**Table 2:** Treatments administered in the study population.

Characteristic	Overall N = 214*^1^*	After CTIO introduction N = 110*^1^*	Before CTIO introduction, N = 104*^1^*	p-value*^2^*
First line treatment				
Carboplatin-etoposide	90 (42.0%)	13 (11.8%)	77 (74.0%)	
Atezolizumab-carboplatin-etoposide	89 (41.6%)	89 (80.9%)	0 (0.0%)	
Cisplatin-etoposide	35 (16.4%)	8 (7.3%)	27 (26.0%)	
Number of first line chemotherapy cycles	4 (3, 6)	4 (4, 4)	5 (2, 6)	
Number of first line immunotherapy cycles	6 (4, 9)	6 (4, 9)		
Thoracic radiotherapy consolidation during first line	15 (7.0%)	13 (11.8%)	2 (1.9%)	0.010
Palliative radiotherapy during first line	61 (28.5%)	32 (29.1%)	29 (27.9%)	>0.9
Further lines	71 (35.9%)	34 (36.2%)	37 (35.6%)	>0.9
Number of further lines				
1	54 (76.1%)	26 (76.5%)	28 (75.7%)	
2	14 (19.7%)	6 (17.6%)	8 (21.6%)	
3	3 (4.2%)	2 (5.9%)	1 (2.7%)	
Second line treatment				
Topotecan	29 (40.8%)	9 (26.5%)	20 (54.1%)	
Lurbinectedin	12 (16.9%)	10 (29.4%)	2 (5.4%)	
Cyclophosphamide-doxorubicin-vincristine	11 (15.5%)	4 (11.8%)	7 (18.9%)	
Other	10 (14.1%)	3 (8.8%)	7 (18.9%)	
Platinum-etoposide rechallenge	8 (11.3%)	8 (23.5%)	0 (0.0%)	
Nivolumab	1 (1.4%)	0 (0.0%)	1 (2.7%)	

^1^ n (%); Median (IQR).

^2^ Pearson’s Chi-squared test.

### 3.2 Real-world patients excluded from immunotherapy treatment

After the introduction of chemo-immunotherapy in clinical practice, eighty-nine patients out of 110 (80.9%) received carboplatin, etoposide and atezolizumab whereas 21 (19.1%) were excluded from chemo-immunotherapy combination and received platinum and etoposide doublet (**Table 2**). The most frequent reason for patients to be considered not eligible for immunotherapy was the need of high dose steroid treatment for symptoms management (12 patients, 57.1%), followed by the presence of active autoimmune disorders (8 patients, 38.1%) (**Table 3**). Two cases of autoimmune disorders were considered as paraneoplastic.

**Table 3:** Reasons for not considering patients eligible for chemo-immunotherapy according to treating physician’s choice.

Reason for not receiving immunotherapy	N=110^1^
Steroid	12 (57.1%)
Autoimmune disease	8 (38.1%)
Performance status	1 (4.8%)
Autoimmune disease	
Ankylosing spondylitis	2 (18.2%)
Addison disease	1 (9.1%)
Autoimmune neuropathy with amphiphysin and SOX1 autoantibodies	1 (9.1%)
Dermatomiositis	1 (9.1%)
Indifferentiated connectivitis	1 (9.1%)
Pulmonary fibrosis	1 (9.1%)
Rheumatoid arthritis	1 (9.1%)
RS3PE syndrome	1 (9.1%)
Systemic sclerosis	1 (9.1%)
Ulcerative colitis	1 (9.1%)

^1^ n (%).

### 3.3 Outcome of the study population, response to treatment and access to further lines treatment

**Figure 1:**
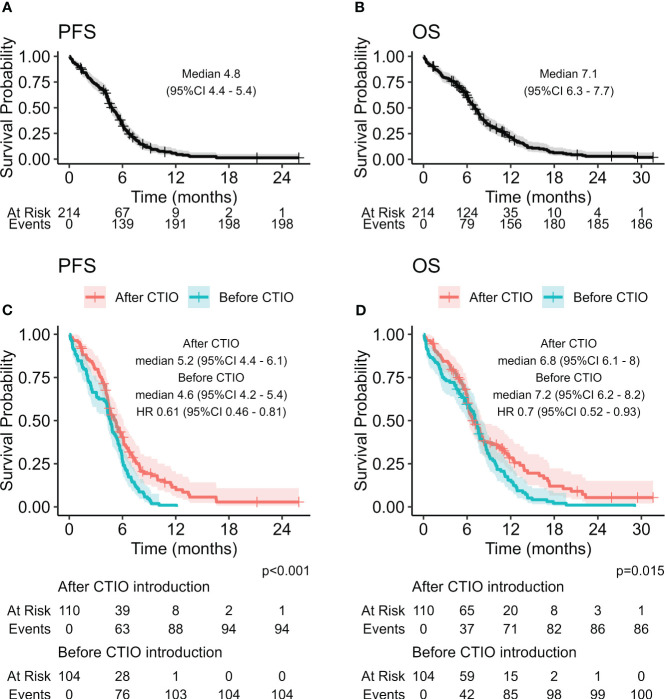
Kaplan-Meier curves estimating PFS **(A)** and OS **(B)** of the overall study population, and comparison of PFS **(C)** and OS **(D)** before and after the introduction of chemo-immunotherapy.

Median PFS of the overall study population was 4.8 months (95% CI: 4.4-5.4), median OS was 7.1 months (95% CI: 6.3-7.7) (**Figure 1**). ORR was 62.7% and DCR was 69.8% (**Table 4**).

**Table 4:** Treatment response in the study population.

Characteristic	Overall, N = 212 [95% CI]*^1,2^*	After CTIO introduction N = 108 [95% CI]*^1,2^*	Before CTIO introduction N = 104 [95% CI]*^1,2^*	p-value*^3^*
RECIST best response				
CR	3 (1.4%)	2 (1.9%)	1 (1.0%)	
PR	130 (61.3%)	73 (67.6%)	57 (54.8%)	
SD	15 (7.1%)	9 (8.3%)	6 (5.8%)	
PD	64 (30.2%)	24 (22.2%)	40 (38.5%)	
ORR	133 (62.7%) [56.1%, 69.0%]	75 (69.4%) [60.2%, 77.3%]	58 (55.8%) [46.2%, 64.9%]	0.055
DCR	148 (69.8%) [63.3%, 75.6%]	84 (77.8%) [69.1%, 84.6%]	64 (61.5%) [51.9%, 70.3%]	0.015

1 n (%).

2 CI, Confidence Interval.

3 Pearson’s Chi-squared test.

When we analyzed the potential impact of clinical factors on outcome, at univariate analysis, ECOG PS 0-1 and no treatment with steroids at baseline were associated with a reduced risk of progression and death, while baseline liver metastases and baseline NLR ≥ 3 were associated with a higher risk of death (**Supplementary Table 1**).

At the data cut-off (October 1, 2023), one hundred and ninety-eight patients (92.5%) experienced disease progression: all patients starting treatment before the introduction of chemo-immunotherapy and 94 out of 110 patients (85.4%) receiving treatment after the introduction of chemoimmunotherapy. The pattern of relapse after first-line treatment is showed in **Supplementary Table 2**.

In the overall study population, seventy-one patients (35.9%) received further lines of treatment, 17 of them (23.9%) received more than one line of treatment after progression (**Table 2**). Most frequent second-line therapy was topotecan (40.8%), while platinum-etoposide rechallenge was administered in 8 cases (11.3%) (**Table 2**).

### 3.4 Comparison of pre-defined clinical indicators before and after the introduction of immunotherapy

Before the introduction of chemo-immunotherapy, 58 patients out of 104 (55.8%) demonstrated an objective radiological response, *versus* 75 out of 108 (69.4%) of those treated afterwards with available radiological evaluation (p = 0.055) (**Table 4**). DCR was 61.5% *versus* 77.8%, respectively (p = 0.015) (**Table 4**).

We compared estimated PFS and OS of the two cohorts of patients and discovered a significant improvement in PFS with HR of 0.61 (95% CI: 0.46-0.81, p < 0.001) (**Figure 1**, **Table 5**) and OS with HR of 0.70 (95% CI: 0.52-0.93, p = 0.015). mPFS was 4.6 months (95%CI: 4.2-5.4) before introduction of chemo-immunotherapy and 5.2 months (95% CI: 4.4-6.1) after the introduction of chemo-immunotherapy, while mOS was 7.2 months (95% CI: 6.2-8.2) and 6.8 months (95% CI: 6.1-8), respectively (**Figure 1**).

**Table 5:** Summary of key outcome indicators comparing outcome before and after introduction of chemo-immunotherapy.

Characteristic	6-month PFS	12-month PFS	12-month OS	18-month OS
Overall	34% (28%, 41%)	5.7% (3.2%, 10%)	22% (16%, 28%)	6.4% (3.5%, 11%)
Group				
After CTIO introduction	40% (32%, 51%)	11% (6.2%, 21%)	28% (20%, 39%)	12% (6.5%, 22%)
Before CTIO introduction	27% (20%, 37%)	1.0% (0.1%, 6.8%)	15% (9.7%, 25%)	2.1% (0.5%, 8.1%)
p-value*^1^*	0.04119	0.0031	0.03169	0.0096

^1^ cloglog fixed-point test.

Although mPFS and mOS were similar in the two cohorts of patients, a significant increase in all the pre-defined clinical indicators was found after the introduction of chemo-immunotherapy (**Table 5**). In particular, 6-month PFS rate increased from 27% (95% CI: 20%-37%) to 40% (95% CI: 32%-51%; p = 0.04); 12-months PFS raised from 1% (95% CI: 0.1%-6.8%) to 11% (95% CI: 6.2%-21%; p = 0.003). 12-month OS rate increased from 15% (95% CI: 9.7%-24%) to 28% (95% CI: 20%-39%; p = 0.03), while 18-month OS was only 2.1% (95% CI: 0.5%-8.1%) before introduction of chemo-immunotherapy and reached 12% afterwards (95% CI: 6.5%-22%; p = 0.009) (**Table 5**, **Figure 1** and **Figure 3**).

### 3.5 Real-life approach and toxicity after the introduction of chemo-immunotherapy in clinical practice

After the introduction of immunotherapy in clinical practice, the median number of platinum-based chemotherapy cycles administered was 4, while the median number of cycles administered before was 5. The median number of atezolizumab administrations received by each patient was 6 (range 1-8) (**Table 2**). A significant increase in the rate of patients receiving thoracic radiotherapy consolidation was recorded (11.8% compared to 1.9%; p = 0.005; **Table 2**), without related G4-5 toxicity.

**Table 6A** summarizes the ten most frequent adverse events recorded and **Table 6B** shows immune-related toxicities experienced by patients included in the analysis. G3-4 toxicities were reported in 31 (28.2%) of patients after the introduction of immunotherapy and 28 (26.9%) before. G5 events took place in two patients in both cohorts (one febrile neutropenia and one sepsis after chemo-immunotherapy introduction and two sepsis before chemo-immunotherapy introduction). Seven patients (7.9%) experienced G3-4 immune-related adverse events (irAEs) and four of them were neurological (**Table 4**). Seven (7.9%) patients discontinued treatment due to irAEs.

**Table 6:** Main adverse events experienced during treatment: the 10 most frequent toxicities **(A)** and immune related toxicities in patients treated with chemo-immunotherapy **(B)**.

A.						
Toxicity	Overall		After CTIO introduction		Before CTIO introduction	
	G1-G2	G3-G4	G1-G2	G3-G4	G1-G2	G3-G4
Anemia	70 (32.7)	18 (8.4)	29 (26.4)	10 (9.1)	41 (39.4)	8 (7.7)
Fatigue	51 (23.8)	4 (1.9)	28 (25.5)	2 (1.8)	23 (22.1)	2 (1.9)
Platelet count decreased	33 (15.4)	12 (5.6)	13 (11.8)	3 (2.7)	20 (19.2)	9 (8.7)
Nausea	19 (8.9)	–	8 (7.3)	–	11 (10.6)	–
Neutrophil count decreased	14 (6.5)	29 (13.6)	7 (6.4)	14 (12.7)	7 (6.7)	15 (14.4)
Constipation	11 (5.1)	–	5 (4.5)	–	6 (5.8)	–
Hypothyroidism	11 (5.1)	–	11 (10.0)	–	–	–
Mucositis oral	10 (4.7)	–	3 (2.7)	–	7 (6.7)	–
Hyperthyroidism	7 (3.3)	–	7 (6.4)	–	–	–
Alanine aminotransferase increased	6 (2.8)	2 (0.9)	5 (4.5)	–	1 (1.0)	2 (1.9)
B.						
**Toxicity**			**G1-G2**		**G3-G4**	
Any toxicity			28 (31.5)		7 (7.9)	
Hypothyroidism			10 (11.2)		–	
Hyperthyroidism			7 (7.9)		–	
Pruritus			5 (5.6)		–	
Alanine aminotransferase increased			3 (3.4)		–	
Aspartate aminotransferase increased			3 (3.4)		–	
Rash maculo-papular			2 (2.2)		–	
Serum amylase increased			2 (2.2)		1 (1.1)	
Adrenal insufficiency			1 (1.1)		–	
Diarrhea			1 (1.1)		1 (1.1)	
Infusion reaction			1 (1.1)		–	
Lipase increased			1 (1.1)		2 (2.2)	
Pneumonitis			1 (1.1)		–	
Rash acneiform			1 (1.1)		–	
Encephalopathy			–		1 (1.1)	
Myalgia			–		1 (1.1)	
Myasthenia gravis			–		1 (1.1)	
Myositis			–		1 (1.1)	

n (%).

**Figure 2:**
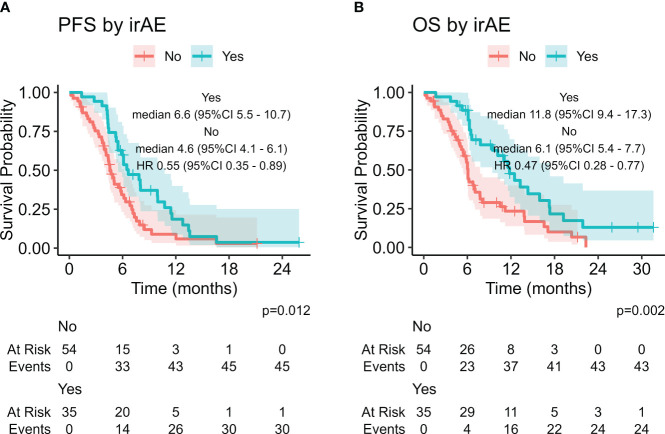
Impact of the presence of immune-related adverse events (irAEs) on PFS **(A)** and OS **(B)**.

The presence of irAE was associated with improved PFS and OS with statistical significance (HR 0.55, 95% CI: 0.35-0.89, p = 0.012 and HR 0.47, 95%CI 0.28-0.77, p = 0.002, respectively, **Figure 2**) . We also considered other potential predictive factors, such as sex, smoke, age at diagnosis, baseline ECOG PS, brain, liver and bone metastases at baseline, NLR, dNLR, PLR, LIPI score and the use of steroids or proton pump inhibitors at baseline: at multivariate analysis ECOG PS 0-1 was the only statistically significant prognostic factor in both after and before chemo-immunotherapy study groups (**Supplementary Table 1**).

The pattern of progression to first line systemic treatment was similar in both study groups (**Supplementary Table 2**).

In patients receiving chemo-immunotherapy multivariate analysis confirmed a significant correlation between baseline steroid treatment and a worse PFS, and a significant correlation between higher baseline ECOG PS and baseline steroid treatment and worse OS (**Supplementary Table 3**).

### 3.6 Hospitalization before and after the introduction of immunotherapy

**Figure 3:**
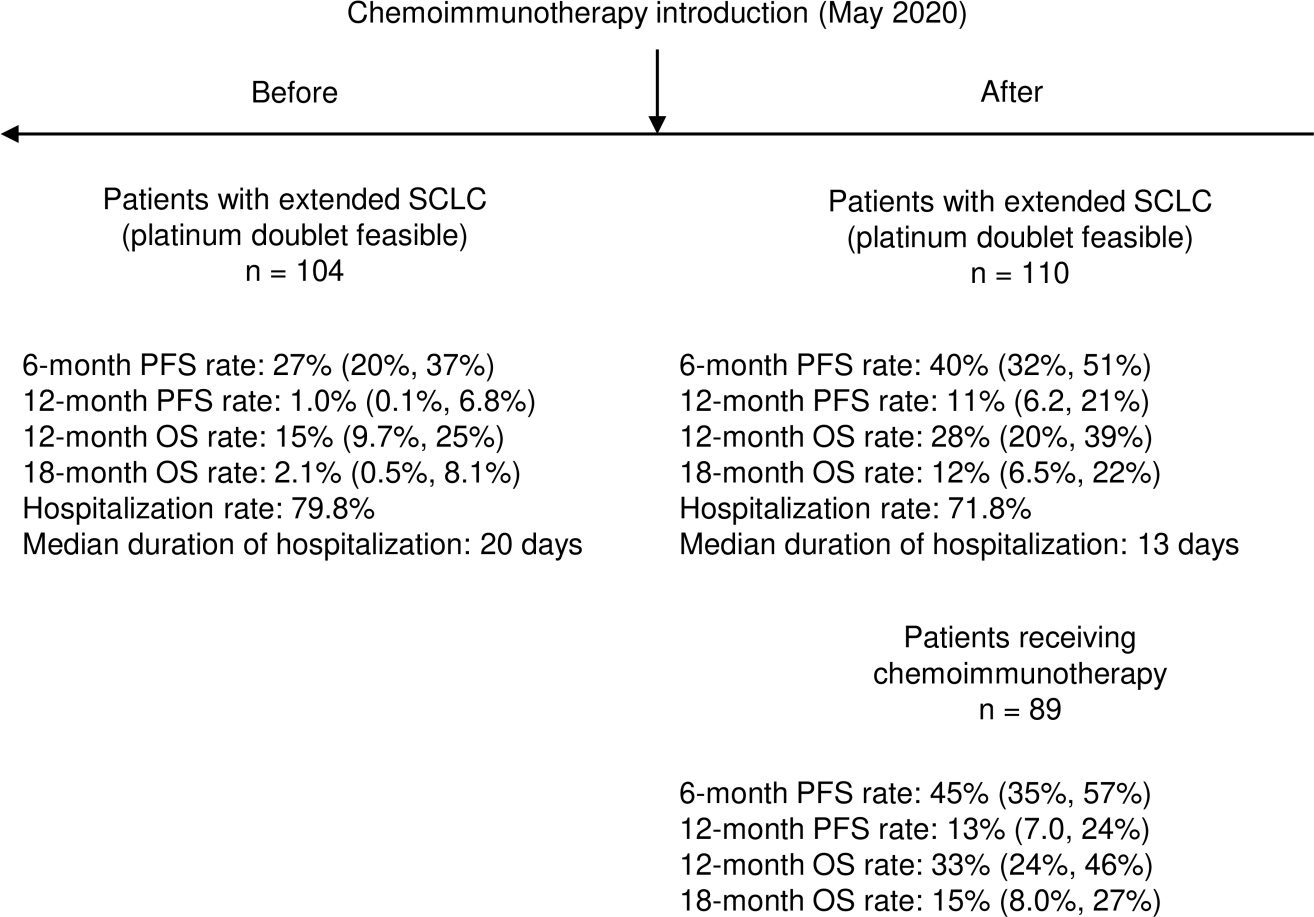
Summary of key results about clinical impact of introduction of chemo-immunotherapy in real-world setting.

To evaluate the impact of the introduction of chemo-immunotherapy on clinical management of patients, we evaluated the rate of hospitalization and the duration of hospitalization during first line treatment (**Table 7**, **Figure 3**). The number of patients requiring hospitalization was 162 (75.7%) in the overall study population and was not significantly different in the two cohorts. Median duration of the hospitalization for each patient was significantly longer before the introduction of chemo-immunotherapy (20 days) compared to after the introduction of chemoimmunotherapy (13 days; p < 0.001) (**Table 7**, **Figure 3**). Reasons for hospitalization are depicted in **Table 7**.

**Table 7:** Comparison of hospitalization days per patient before and after the introduction of chemo-immunotherapy.

Characteristic	Overall N = 214*^1^*	After CTIO introduction N = 110*^1^*	Before CTIO introduction N = 104*^1^*	p-value*^2^*
Hospitalization	162 (75.7%)	79 (71.8%)	83 (79.8%)	0.2
Total days of hospitalization	16 (8, 24)	13 (6, 20)	20 (11, 28)	<0.001
Hospitalization for treatment administration	139 (85.8%)	64 (81.0%)	75 (90.4%)	0.088
Total days of hospitalization for treatment administration	12 (5, 18)	12 (4, 14)	12 (6, 18)	0.2
Hospitalization for toxicity	29 (17.9%)	13 (16.5%)	16 (19.3%)	0.6
Total days of hospitalization for toxicity	8 (5, 15)	8 (4, 11)	8 (6, 16)	0.5

Only hospitalizations occurred from first line therapy start to first line therapy progression were considered.

^1^ n (%); Median (IQR).

^2^ Pearson’s Chi-squared test; Wilcoxon rank sum test.

## 4 Discussion

The addition of immunotherapy to first-line chemotherapy for extended SCLC has been approved for clinical practice according to randomized clinical trials (10, 11), but its actual impact in real-world setting is thought to be impaired by clinical features of RW patients, often presenting highly symptomatic and with several comorbidities related to age and smoking habits (21). In the present study we analyzed the impact of introduction of chemo-immunotherapy in clinical practice.

We noticed that after the introduction of chemo-immunotherapy, the median dosage of steroids administered at diagnosis decreased significantly, even though clinical features of the study populations were comparable. This indicates how immunotherapy has changed our clinical attitude in managing symptoms.

On the other hand, we noticed an increase in the rate of patients receiving consolidation radiotherapy after the introduction of chemo-immunotherapy. We can speculate that the attitude might be mainly related to increased depth of responses induced by chemo-immunotherapy. The role of consolidation thoracic radiotherapy in extended SCLC has been demonstrated in CREST study and some data already exist concerning its role in patients treated with chemo-immunotherapy, although increased incidence of treatment-related pneumonitis is observed (27–30). Pneumonitis observed in patients treated sequentially seem to be mainly slightly symptomatic and manageable, while TREASURE study of concomitant atezolizumab plus consolidation radiotherapy was closed permanently due to safety alert related mainly to severe pneumonitis, including treatment-related deaths (27, 31).

Other indirect changes induced in clinical practice are the reduced number of chemotherapy cycles and, more importantly, the decreased number of days of hospitalization per patient. This might be due to both reduced chemotherapy administration and to the decreased incidence and severity of adverse events. Anyway, hospitalization days were chosen as pre-defined indicators, since they might represent a surrogate parameter for both financial toxicity and quality of life.

Finally, we decided to evaluate clinical impact of the introduction of chemo-immunotherapy in clinical practice by evaluating changes in clinical outcome endpoints between two study population (before and after the introduction of ICIs), including also patients who were not eligible for immunotherapy, in order to conservatively reduce the impact of clinical selection bias. The analysis demonstrates a statistically significant improvement in PFS and OS and, more importantly, a clinically meaningful increase in the rate of patients free of progression and alive at pre-defined timepoints. The results confirm the likelihood of relatively long survivorship led by the addition of immunotherapy to chemotherapy in extended SCLC. Recently, long-term survival data obtained among patients treated with carboplatin etoposide and atezolizumab were presented in IMbrella A study and an exceptional 5-year OS rate of 12% was reported, while 3-year OS rate reported for chemotherapy plus durvalumab was 17.6% (32, 33).

In our series, the follow-up for patients included after the introduction of chemo-immunotherapy is likely to be too short to underline the tails of the curves, that represent the most clinically meaningful features of clinical impact of immunotherapy. In our series median follow-up was 25.9 months for patients treated after the introduction of chemo-immunotherapy, while median follow-up was 39.4 months for patients treated with durvalumab in updated OS analysis of CASPIAN trial and 59.4 months for patients treated with atezolizumab in IMbrella A observational study (32, 33). Anyway, 12-months PFS increased from 1 to 11% and 18-month OS from 2 to 12% in a RW setting, clearly showing the clinical impact of chemo-immunotherapy in real-world clinical setting.

In this context, the role of immunotherapy seems to be consistently confirmed in real-world setting and even in ECOG PS 2-3 patients in line with previously reported series mainly in Asian population (34–42).

The main strength points of our study are related to the multicentric nature of the data collection, thus including both Academic and Community General Hospitals, and the relatively high number of patients included, when compared to previously published reports. In addition, as over-mentioned, the inclusion of patients excluded from chemo-immunotherapy after the introduction of ICIs for SCLC permits to minimize clinical selection bias and underlines the actual impact of immunotherapy in RW setting. On the other side, the main weak points are retrospective nature of the study, relatively short follow-up for the most recent cohort of patients.

In conclusion, although considering the depicted limitations, our study depicts a large Caucasian multicentric RW population and demonstrates the improvement in outcome endpoints induced by the introduction of immunotherapy.

## Data availability statement

The raw data supporting the conclusions of this article will be made available by the authors, without undue reservation.

## Ethics statement

The studies involving humans were approved by Istituto Oncologico Veneto IOV-IRCCS. The studies were conducted in accordance with the local legislation and institutional requirements. Written informed consent for participation was not required from the participants or the participants’ legal guardians/next of kin in accordance with the national legislation and institutional requirements.

## Author contributions

LB: Conceptualization, Data curation, Investigation, Resources, Supervision, Writing – original draft, Writing – review & editing. LC: Conceptualization, Resources, Writing – review & editing. ADM: Data curation, Formal analysis, Methodology, Resources, Visualization, Writing – original draft, Writing – review & editing. AP: Resources, Writing – review & editing. LCB: Data curation, Resources, Writing – review & editing. MD: Data curation, Resources, Writing – review & editing. SF: Resources, Writing – review & editing. GS: Resources, Writing – review & editing. AF: Resources, Writing – review & editing. GP: Resources, Writing – review & editing. PM: Resources, Supervision, Writing – review & editing. GA: Resources, Supervision, Writing – review & editing. VG: Supervision, Writing – review & editing.

## Supplementary material

Supplementary Figure 1 Study design and patient disposition.

Supplementary Figure 2 Comparison of PFS **(A)** and OS **(B)** between patients receiving chemoimmunotherapy (CTIO) and chemotherapy (CT).
