# Supplementary figures and images for: Real-world impact of the introduction of chemo-immunotherapy in extended small cell lung cancer: a multicentric analysis
Source: Front Immunol. 2024 Jan 22;15:1353889. doi: 10.3389/fimmu.2024.1353889 (PMC10845350; doi:10.3389/fimmu.2024.1353889)

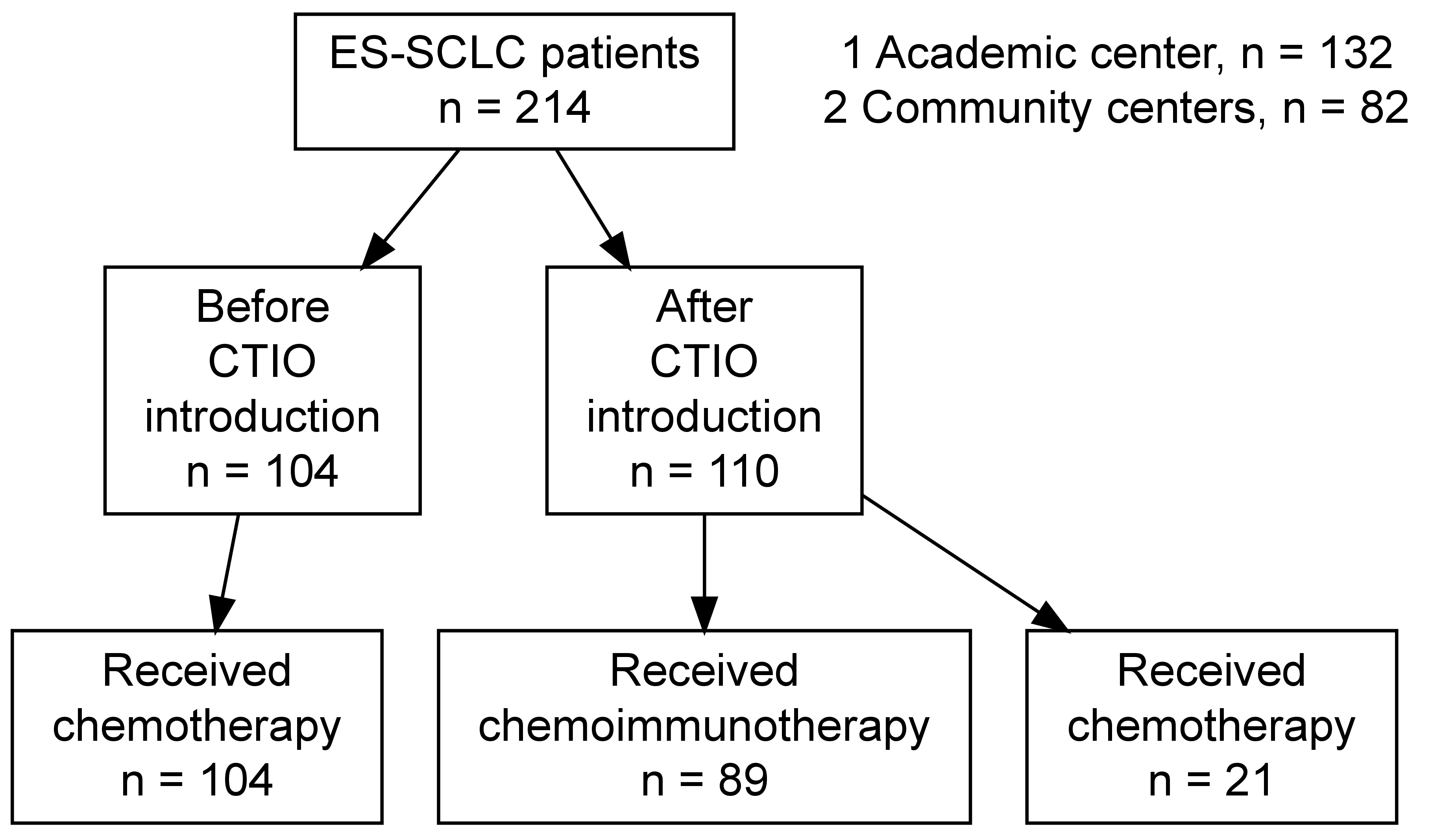

Supplement: Supplementary Figure 1 — Study design and patient disposition. [file Image_1.jpeg]

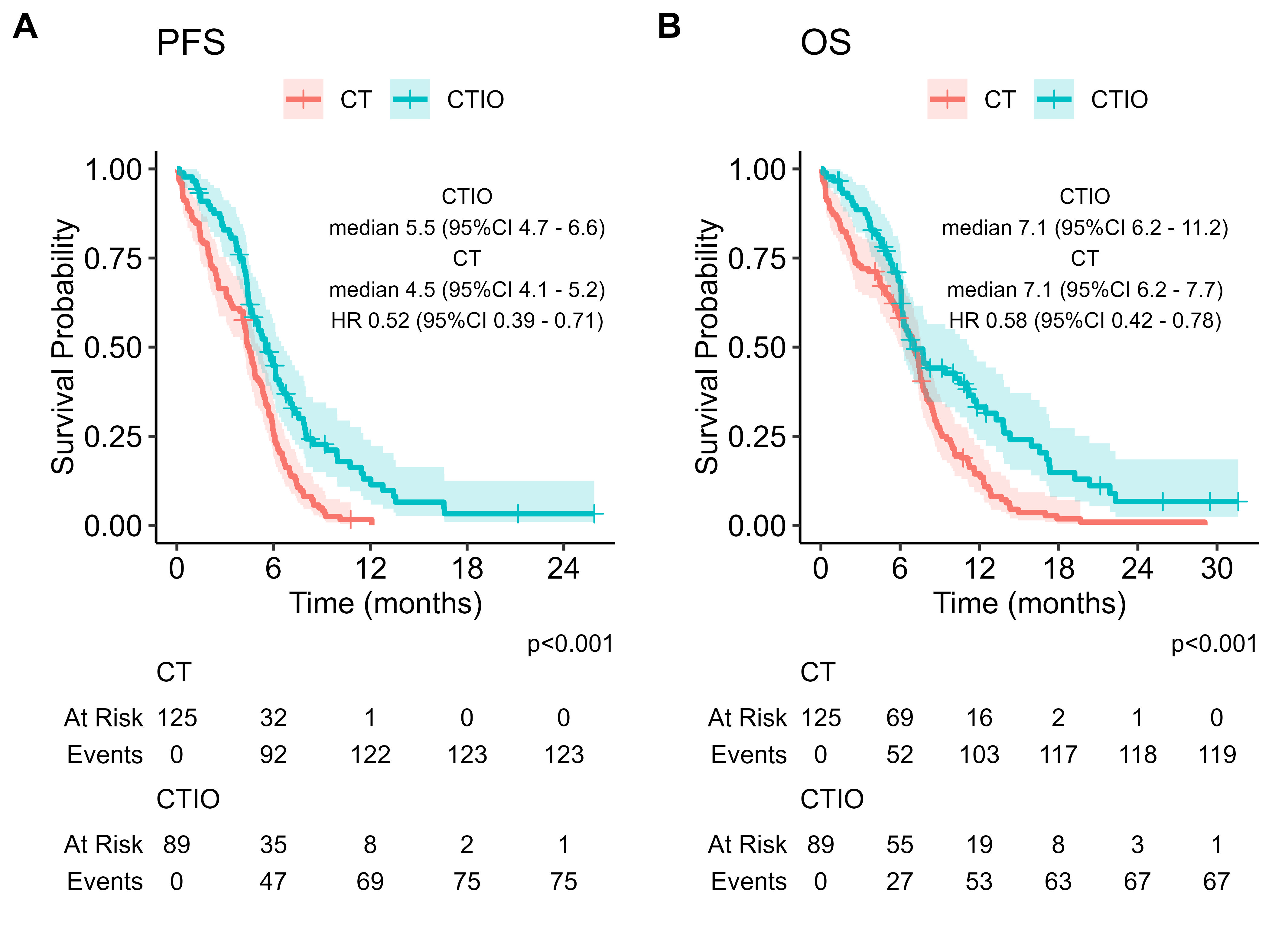

Supplement: Supplementary Figure 2 — Comparison of PFS (A) and OS (B) between patients receiving chemoimmunotherapy (CTIO) and chemotherapy (CT). [file Image_2.jpeg]
