# Supplementary material for: Real-world impact of the introduction of chemo-immunotherapy in extended small cell lung cancer: a multicentric analysis
Source: Front Immunol. 2024 Jan 22;15:1353889. doi: 10.3389/fimmu.2024.1353889 (PMC10845350; doi:10.3389/fimmu.2024.1353889)
Supplement: Supplementary file 3 [file Table_1.docx]

Supplementary Table 1. Distribution of main potential prognostic factors in the overall study population and the two cohorts of patients and Cox regression analysis for association with progression-free survival and overall survival

| **Characteristic** | **Univariate PFS** | | | | | | **Multivariate PFS** | | | **Univariate OS** | | | | **Multivariate OS** | | |
| --- | --- | --- | --- | --- | --- | --- | --- | --- | --- | --- | --- | --- | --- | --- | --- | --- |
|  | **N** | **HR***^1^* | **95% CI***^1^* | **p-value** | | **HR***^1^* | | **95% CI***^1^* | **p-value** | **N** | **HR***^1^* | **95% CI***^1^* | **p-value** | **HR***^1^* | **95% CI***^1^* | **p-value** |
| Sex | 214 |  |  | |  | |  |  |  | 214 |  |  |  |  |  |  |
| Female |  | — | — | |  | |  |  |  |  | — | — |  |  |  |  |
| Male |  | 0.86 | 0.65, 1.16 | | 0.3 | |  |  |  |  | 0.96 | 0.71, 1.29 | 0.8 |  |  |  |
| Smoking category | 209 |  |  | |  | |  |  |  | 209 |  |  |  |  |  |  |
| Actual |  | — | — | |  | |  |  |  |  | — | — |  |  |  |  |
| Former or never |  | 0.84 | 0.62, 1.13 | | 0.2 | |  |  |  |  | 0.88 | 0.65, 1.19 | 0.4 |  |  |  |
| Age at diagnosis | 214 |  |  | |  | |  |  |  | 214 |  |  |  |  |  |  |
| <= 70 |  | — | — | |  | |  |  |  |  | — | — |  |  |  |  |
| > 70 |  | 1.12 | 0.85, 1.49 | | 0.4 | |  |  |  |  | 1.15 | 0.86, 1.54 | 0.3 |  |  |  |
| Baseline ECOG PS | 214 |  |  | |  | |  |  |  | 214 |  |  |  |  |  |  |
| > 1 |  | — | — | |  | | — | — |  |  | — | — |  | — | — |  |
| 0-1 |  | 0.72 | 0.54, 0.97 | | 0.029 | | 0.79 | 0.58, 1.07 | 0.13 |  | 0.52 | 0.38, 0.71 | <0.001 | 0.84 | 0.59, 1.19 | 0.3 |
| Brain metastasis at baseline | 214 |  |  | |  | |  |  |  | 214 |  |  |  |  |  |  |
| No |  | — | — | |  | |  |  |  |  | — | — |  |  |  |  |
| Yes |  | 1.22 | 0.87, 1.72 | | 0.2 | |  |  |  |  | 1.40 | 0.99, 1.98 | 0.060 |  |  |  |
| Bone metastasis at baseline | 214 |  |  | |  | |  |  |  | 214 |  |  |  |  |  |  |
| No |  | — | — | |  | |  |  |  |  | — | — |  |  |  |  |
| Yes |  | 0.87 | 0.63, 1.20 | | 0.4 | |  |  |  |  | 1.04 | 0.75, 1.45 | 0.8 |  |  |  |
| Liver metastasis at baseline | 214 |  |  | |  | |  |  |  | 214 |  |  |  |  |  |  |
| No |  | — | — | |  | |  |  |  |  | — | — |  | — | — |  |
| Yes |  | 1.23 | 0.93, 1.63 | | 0.14 | |  |  |  |  | 1.49 | 1.11, 1.99 | 0.008 | 1.08 | 0.78, 1.48 | 0.6 |
| NLR at baseline | 184 |  |  | |  | |  |  |  | 184 |  |  |  |  |  |  |
| < 3 |  | — | — | |  | |  |  |  |  | — | — |  | — | — |  |
| >= 3 |  | 1.29 | 0.93, 1.77 | | 0.12 | |  |  |  |  | 1.62 | 1.15, 2.27 | 0.005 | 1.13 | 0.81, 1.58 | 0.5 |
| dNLR baseline | 206 |  |  | |  | |  |  |  | 206 |  |  |  |  |  |  |
| < 3 |  | — | — | |  | |  |  |  |  | — | — |  |  |  |  |
| >= 3 |  | 1.13 | 0.83, 1.53 | | 0.4 | |  |  |  |  | 1.20 | 0.88, 1.63 | 0.2 |  |  |  |
| PLR baseline | 184 |  |  | |  | |  |  |  | 184 |  |  |  |  |  |  |
| < 180 |  | — | — | |  | |  |  |  |  | — | — |  |  |  |  |
| >= 180 |  | 1.08 | 0.80, 1.46 | | 0.6 | |  |  |  |  | 1.30 | 0.94, 1.78 | 0.11 |  |  |  |
| LIPI category at baseline | 106 |  |  | |  | |  |  |  | 106 |  |  |  |  |  |  |
| Good-Intermediate |  | — | — | |  | |  |  |  |  | — | — |  |  |  |  |
| Poor |  | 0.93 | 0.59, 1.49 | | 0.8 | |  |  |  |  | 1.00 | 0.62, 1.62 | >0.9 |  |  |  |
| Baseline steroid | 211 |  |  | |  | |  |  |  | 211 |  |  |  |  |  |  |
| No |  | — | — | |  | | — | — |  |  | — | — |  | — | — |  |
| Yes |  | 1.60 | 1.20, 2.14 | | 0.001 | | 1.55 | 1.16, 2.07 | 0.003 |  | 2.01 | 1.49, 2.72 | <0.001 | 1.37 | 1.00, 1.88 | 0.053 |
| *^1^* HR = Hazard Ratio, CI = Confidence Interval | | | | | | | | | | | | | | | | |
|  |  |  |  | |  | |  |  |  |  |  |  |  |  |  |  |
| **After CTIO introduction** |  |  |  | |  | |  |  |  |  |  |  |  |  |  |  |
|  |  |  |  | |  | |  |  |  |  |  |  |  |  |  |  |
| **Characteristic** | **Univariate PFS** | | | | | | **Multivariate PFS** | | | **Univariate OS** | | | | **Multivariate OS** | | |
|  | **N** | **HR***^1^* | **95% CI***^1^* | | **p-value** | | **HR***^1^* | **95% CI***^1^* | **p-value** | **N** | **HR***^1^* | **95% CI***^1^* | **p-value** | **HR***^1^* | **95% CI***^1^* | **p-value** |
| Sex | 110 |  |  | |  | |  |  |  | 110 |  |  |  |  |  |  |
| Female |  | — | — | |  | |  |  |  |  | — | — |  |  |  |  |
| Male |  | 0.95 | 0.62, 1.44 | | 0.8 | |  |  |  |  | 1.00 | 0.65, 1.55 | >0.9 |  |  |  |
| Smoking category | 110 |  |  | |  | |  |  |  | 110 |  |  |  |  |  |  |
| Actual |  | — | — | |  | | — | — |  |  | — | — |  |  |  |  |
| Former or never |  | 0.63 | 0.41, 0.99 | | 0.043 | | 0.81 | 0.60, 1.10 | 0.2 |  | 0.65 | 0.41, 1.02 | 0.062 |  |  |  |
| Age at diagnosis 70 | 110 |  |  | |  | |  |  |  | 110 |  |  |  |  |  |  |
| <= 70 |  | — | — | |  | |  |  |  |  | — | — |  |  |  |  |
| > 70 |  | 0.96 | 0.64, 1.46 | | 0.9 | |  |  |  |  | 1.22 | 0.80, 1.87 | 0.4 |  |  |  |
| Baseline ECOG PS | 110 |  |  | |  | |  |  |  | 110 |  |  |  |  |  |  |
| > 1 |  | — | — | |  | | — | — |  |  | — | — |  | — | — |  |
| 0-1 |  | 0.62 | 0.41, 0.95 | | 0.029 | | 0.80 | 0.59, 1.08 | 0.15 |  | 0.47 | 0.30, 0.73 | <0.001 | 0.62 | 0.43, 0.88 | 0.007 |
| Brain metastasis at baseline | 110 |  |  | |  | |  |  |  | 110 |  |  |  |  |  |  |
| No |  | — | — | |  | |  |  |  |  | — | — |  |  |  |  |
| Yes |  | 1.26 | 0.78, 2.04 | | 0.3 | |  |  |  |  | 1.31 | 0.80, 2.15 | 0.3 |  |  |  |
| Bone metastasis at baseline | 110 |  |  | |  | |  |  |  | 110 |  |  |  |  |  |  |
| No |  | — | — | |  | |  |  |  |  | — | — |  |  |  |  |
| Yes |  | 0.95 | 0.60, 1.51 | | 0.8 | |  |  |  |  | 1.01 | 0.62, 1.65 | >0.9 |  |  |  |
| Liver metastasis at baseline | 110 |  |  | |  | |  |  |  | 110 |  |  |  |  |  |  |
| No |  | — | — | |  | |  |  |  |  | — | — |  |  |  |  |
| Yes |  | 1.29 | 0.86, 1.95 | | 0.2 | |  |  |  |  | 1.39 | 0.90, 2.13 | 0.14 |  |  |  |
| NLR at baseline | 101 |  |  | |  | |  |  |  | 101 |  |  |  |  |  |  |
| < 3 |  | — | — | |  | |  |  |  |  | — | — |  | — | — |  |
| >= 3 |  | 1.40 | 0.87, 2.25 | | 0.2 | |  |  |  |  | 1.79 | 1.07, 3.00 | 0.026 | 1.23 | 0.86, 1.76 | 0.3 |
| dNLR at baseline | 109 |  |  | |  | |  |  |  | 109 |  |  |  |  |  |  |
| < 3 |  | — | — | |  | |  |  |  |  | — | — |  |  |  |  |
| >= 3 |  | 1.20 | 0.79, 1.83 | | 0.4 | |  |  |  |  | 1.24 | 0.81, 1.92 | 0.3 |  |  |  |
| PLR at baseline | 101 |  |  | |  | |  |  |  | 101 |  |  |  |  |  |  |
| < 180 |  | — | — | |  | |  |  |  |  | — | — |  |  |  |  |
| >= 180 |  | 1.25 | 0.81, 1.92 | | 0.3 | |  |  |  |  | 1.56 | 0.98, 2.48 | 0.061 |  |  |  |
| LIPI category at baseline | 57 |  |  | |  | |  |  |  | 57 |  |  |  |  |  |  |
| Good-Intermediate |  | — | — | |  | |  |  |  |  | — | — |  |  |  |  |
| Poor |  | 0.89 | 0.48, 1.65 | | 0.7 | |  |  |  |  | 0.85 | 0.44, 1.65 | 0.6 |  |  |  |
| Baseline steroid | 110 |  |  | |  | |  |  |  | 110 |  |  |  |  |  |  |
| No |  | — | — | |  | | — | — |  |  | — | — |  | — | — |  |
| Yes |  | 2.40 | 1.57, 3.69 | | <0.001 | | 1.36 | 0.97, 1.91 | 0.075 |  | 2.70 | 1.71, 4.25 | <0.001 | 1.41 | 0.98, 2.05 | 0.067 |
| Baseline PPI | 110 |  |  | |  | |  |  |  | 110 |  |  |  |  |  |  |
| No |  | — | — | |  | | — | — |  |  | — | — |  | — | — |  |
| Yes |  | 1.92 | 1.18, 3.11 | | 0.008 | | 1.37 | 0.92, 2.03 | 0.13 |  | 2.61 | 1.51, 4.51 | <0.001 | 1.44 | 0.93, 2.23 | 0.10 |
| *^1^* HR = Hazard Ratio, CI = Confidence Interval | | | | | | | | | | | | | | | | |
|  |  |  |  | |  | |  |  |  |  |  |  |  |  |  |  |
| **Before CTIO introduction** |  |  |  | |  | |  |  |  |  |  |  |  |  |  |  |

| **Characteristic** | **Univariate PFS** | | | | **Multivariate PFS** | | | **Univariate OS** | | | | **Multivariate OS** | | |
| --- | --- | --- | --- | --- | --- | --- | --- | --- | --- | --- | --- | --- | --- | --- |
|  | **N** | **HR***^1^* | **95% CI***^1^* | **p-value** | **HR***^1^* | **95% CI***^1^* | **p-value** | **N** | **HR***^1^* | **95% CI***^1^* | **p-value** | **HR***^1^* | **95% CI***^1^* | **p-value** |
| Sex | 104 |  |  |  |  |  |  | 104 |  |  |  |  |  |  |
| Female |  | — | — |  |  |  |  |  | — | — |  |  |  |  |
| Male |  | 0.76 | 0.51, 1.14 | 0.2 |  |  |  |  | 0.92 | 0.60, 1.41 | 0.7 |  |  |  |
| Smoke | 99 |  |  |  |  |  |  | 99 |  |  |  |  |  |  |
| Actual |  | — | — |  |  |  |  |  | — | — |  |  |  |  |
| Former-Never |  | 1.22 | 0.81, 1.84 | 0.3 |  |  |  |  | 1.42 | 0.92, 2.18 | 0.11 |  |  |  |
| Age at diagnosis | 104 |  |  |  |  |  |  | 104 |  |  |  |  |  |  |
| <= 70 |  | — | — |  |  |  |  |  | — | — |  |  |  |  |
| > 70 |  | 1.47 | 0.99, 2.18 | 0.059 |  |  |  |  | 1.05 | 0.71, 1.57 | 0.8 |  |  |  |
| Baseline ECOG PS | 104 |  |  |  |  |  |  | 104 |  |  |  |  |  |  |
| > 1 |  | — | — |  |  |  |  |  | — | — |  | — | — |  |
| 0-1 |  | 0.84 | 0.56, 1.28 | 0.4 |  |  |  |  | 0.60 | 0.39, 0.92 | 0.019 | 0.64 | 0.45, 0.92 | 0.017 |
| Brain metastases at baseline | 104 |  |  |  |  |  |  | 104 |  |  |  |  |  |  |
| No |  | — | — |  |  |  |  |  | — | — |  | — | — |  |
| Yes |  | 1.33 | 0.82, 2.15 | 0.3 |  |  |  |  | 1.82 | 1.10, 3.01 | 0.020 | 1.25 | 0.83, 1.89 | 0.3 |
| Bone metastases at baseline | 104 |  |  |  |  |  |  | 104 |  |  |  |  |  |  |
| No |  | — | — |  |  |  |  |  | — | — |  |  |  |  |
| Yes |  | 0.79 | 0.51, 1.23 | 0.3 |  |  |  |  | 1.01 | 0.65, 1.58 | >0.9 |  |  |  |
| Liver metastases at baseline | 104 |  |  |  |  |  |  | 104 |  |  |  |  |  |  |
| No |  | — | — |  |  |  |  |  | — | — |  | — | — |  |
| Yes |  | 1.20 | 0.81, 1.76 | 0.4 |  |  |  |  | 1.67 | 1.11, 2.51 | 0.014 | 1.38 | 0.99, 1.92 | 0.056 |
| NLR at baseline | 83 |  |  |  |  |  |  | 83 |  |  |  |  |  |  |
| < 3 |  | — | — |  |  |  |  |  | — | — |  | — | — |  |
| >= 3 |  | 1.27 | 0.81, 1.97 | 0.3 |  |  |  |  | 1.67 | 1.04, 2.68 | 0.034 | 1.42 | 0.99, 2.03 | 0.056 |
| dNLR at baseline | 97 |  |  |  |  |  |  | 97 |  |  |  |  |  |  |
| < 3 |  | — | — |  |  |  |  |  | — | — |  |  |  |  |
| >= 3 |  | 1.09 | 0.69, 1.71 | 0.7 |  |  |  |  | 1.29 | 0.82, 2.02 | 0.3 |  |  |  |
| PLR at baseline | 83 |  |  |  |  |  |  | 83 |  |  |  |  |  |  |
| < 180 |  | — | — |  |  |  |  |  | — | — |  |  |  |  |
| >= 180 |  | 0.89 | 0.57, 1.38 | 0.6 |  |  |  |  | 1.10 | 0.71, 1.71 | 0.7 |  |  |  |
| LIPI at baseline | 49 |  |  |  |  |  |  | 49 |  |  |  |  |  |  |
| Good-Intermediate |  | — | — |  |  |  |  |  | — | — |  |  |  |  |
| Poor |  | 1.34 | 0.64, 2.81 | 0.4 |  |  |  |  | 1.36 | 0.66, 2.77 | 0.4 |  |  |  |
| Baseline steroid | 101 |  |  |  |  |  |  | 101 |  |  |  |  |  |  |
| No |  | — | — |  |  |  |  |  | — | — |  |  |  |  |
| Yes |  | 0.94 | 0.63, 1.40 | 0.8 |  |  |  |  | 1.45 | 0.96, 2.18 | 0.077 |  |  |  |
| Baseline_PPI | 101 |  |  |  |  |  |  | 101 |  |  |  |  |  |  |
| No |  | — | — |  |  |  |  |  | — | — |  |  |  |  |
| Yes |  | 0.92 | 0.57, 1.48 | 0.7 |  |  |  |  | 1.09 | 0.67, 1.78 | 0.7 |  |  |  |
| *^1^* HR = Hazard Ratio, CI = Confidence Interval | | | | | | | | | | | | | | |

Supplementary Table 2. Pattern of relapse after first-line treatment for extended small cell lung cancer

| **Characteristic** | **Overall**, N = 160*^1^* | **After CTIO introduction**  N = 78*^1^* | **Before CTIO introduction**, N = 82*^1^* | **p-value***^2^* |
| --- | --- | --- | --- | --- |
| Intrathoracic | 32 (20%) | 14 (18%) | 18 (22%) | 0.5 |
| Systemic | 141 (88%) | 68 (87%) | 73 (89%) | 0.7 |
| Brain | 60 (38%) | 29 (37%) | 31 (38%) | >0.9 |
| Bone | 31 (19%) | 15 (19%) | 16 (20%) | >0.9 |
| Liver | 61 (38%) | 30 (38%) | 31 (38%) | >0.9 |
| Pleura | 1 (0.6%) | 1 (1.3%) | 0 (0%) | 0.5 |
| Distant_nodes | 32 (20%) | 14 (18%) | 18 (22%) | 0.5 |
| Soft_tissues | 7 (4.4%) | 3 (3.8%) | 4 (4.9%) | >0.9 |
| Adrenal | 13 (8.1%) | 7 (9.0%) | 6 (7.3%) | 0.7 |
| Peritoneum | 1 (0.6%) | 1 (1.3%) | 0 (0%) | 0.5 |
| *^1^* n (%) | | | | |
| *^2^* Pearson’s Chi-squared test; Fisher’s exact test | | | | |

Supplementary Table 3. Impact of potential predictive markers for sensitivity to immunotherapy on progression-free survival and overall survival of patients treated with chemo-immunotherapy

| **Characteristic** | **Univariate PFS** | | | |  |  |  | **Multivariate PFS** | | | **Univariate OS** | | | | **Multivariate OS** | | |
| --- | --- | --- | --- | --- | --- | --- | --- | --- | --- | --- | --- | --- | --- | --- | --- | --- | --- |
|  | **N** | **HR***^1^* | **95% CI***^1^* | **p-value** |  |  |  | **HR***^1^* | **95% CI***^1^* | **p-value** | **N** | **HR***^1^* | **95% CI***^1^* | **p-value** | **HR***^1^* | **95% CI***^1^* | **p-value** |
| Sex | 89 |  |  |  |  |  |  |  |  |  | 89 |  |  |  |  |  |  |
| Female |  | — | — |  |  |  |  |  |  |  |  | — | — |  |  |  |  |
| Male |  | 1.04 | 0.65, 1.67 | 0.9 |  |  |  |  |  |  |  | 1.15 | 0.70, 1.89 | 0.6 |  |  |  |
| Smoke | 89 |  |  |  |  |  |  |  |  |  | 89 |  |  |  |  |  |  |
| Actual |  | — | — |  |  |  |  | — | — |  |  | — | — |  | — | — |  |
| Former-Never |  | 0.56 | 0.34, 0.93 | 0.026 |  |  |  | 0.84 | 0.62, 1.14 | 0.3 |  | 0.58 | 0.34, 0.98 | 0.043 | 0.84 | 0.60, 1.17 | 0.3 |
| Age at diagnosis | 89 |  |  |  |  |  |  |  |  |  | 89 |  |  |  |  |  |  |
| <= 70 |  | — | — |  |  |  |  |  |  |  |  | — | — |  |  |  |  |
| > 70 |  | 0.82 | 0.51, 1.31 | 0.4 |  |  |  |  |  |  |  | 1.18 | 0.73, 1.92 | 0.5 |  |  |  |
| Baseline ECOG PS | 89 |  |  |  |  |  |  |  |  |  | 89 |  |  |  |  |  |  |
| > 1 |  | — | — |  |  |  |  | — | — |  |  | — | — |  | — | — |  |
| 0-1 |  | 0.61 | 0.37, 0.99 | 0.046 |  |  |  | 0.81 | 0.60, 1.10 | 0.2 |  | 0.47 | 0.28, 0.80 | 0.005 | 0.62 | 0.43, 0.88 | 0.008 |
| Brain metastases at baseline | 89 |  |  |  |  |  |  |  |  |  | 89 |  |  |  |  |  |  |
| No |  | — | — |  |  |  |  |  |  |  |  | — | — |  |  |  |  |
| Yes |  | 1.04 | 0.59, 1.84 | 0.9 |  |  |  |  |  |  |  | 1.16 | 0.64, 2.11 | 0.6 |  |  |  |
| Bone metastases at baseline | 89 |  |  |  |  |  |  |  |  |  | 89 |  |  |  |  |  |  |
| No |  | — | — |  |  |  |  |  |  |  |  | — | — |  |  |  |  |
| Yes |  | 0.90 | 0.54, 1.52 | 0.7 |  |  |  |  |  |  |  | 0.96 | 0.54, 1.71 | >0.9 |  |  |  |
| Liver metastases at baseline | 89 |  |  |  |  |  |  |  |  |  | 89 |  |  |  |  |  |  |
| No |  | — | — |  |  |  |  |  |  |  |  | — | — |  |  |  |  |
| Yes |  | 1.53 | 0.97, 2.42 | 0.069 |  |  |  |  |  |  |  | 1.62 | 0.99, 2.64 | 0.054 |  |  |  |
| NLR at baseline | 81 |  |  |  |  |  |  |  |  |  | 81 |  |  |  |  |  |  |
| < 3 |  | — | — |  |  |  |  |  |  |  |  | — | — |  | — | — |  |
| >= 3 |  | 1.38 | 0.82, 2.32 | 0.2 |  |  |  |  |  |  |  | 1.78 | 1.01, 3.14 | 0.047 | 1.14 | 0.75, 1.73 | 0.5 |
| NLR at baseline | 88 |  |  |  |  |  |  |  |  |  | 88 |  |  |  |  |  |  |
| < 3 |  | — | — |  |  |  |  |  |  |  |  | — | — |  |  |  |  |
| >= 3 |  | 1.13 | 0.70, 1.83 | 0.6 |  |  |  |  |  |  |  | 1.16 | 0.71, 1.90 | 0.6 |  |  |  |
| PLR at baseline | 81 |  |  |  |  |  |  |  |  |  | 81 |  |  |  |  |  |  |
| < 180 |  | — | — |  |  |  |  |  |  |  |  | — | — |  | — | — |  |
| >= 180 |  | 1.43 | 0.88, 2.33 | 0.15 |  |  |  |  |  |  |  | 1.74 | 1.01, 2.99 | 0.044 | 1.18 | 0.81, 1.72 | 0.4 |
| LIPI at baseline | 50 |  |  |  |  |  |  |  |  |  | 50 |  |  |  |  |  |  |
| Good-Intermediate |  | — | — |  |  |  |  |  |  |  |  | — | — |  |  |  |  |
| Poor |  | 1.01 | 0.52, 1.96 | >0.9 |  |  |  |  |  |  |  | 0.91 | 0.45, 1.87 | 0.8 |  |  |  |
| Baseline steroid | 89 |  |  |  |  |  |  |  |  |  | 89 |  |  |  |  |  |  |
| No |  | — | — |  |  |  |  | — | — |  |  | — | — |  | — | — |  |
| Yes |  | 2.12 | 1.33, 3.39 | 0.002 |  |  |  | 1.57 | 1.17, 2.11 | 0.003 |  | 2.40 | 1.46, 3.96 | <0.001 | 1.45 | 1.00, 2.11 | 0.049 |
| Baseline PPI | 89 |  |  |  |  |  |  |  |  |  | 89 |  |  |  |  |  |  |
| No |  | — | — |  |  |  |  |  |  |  |  | — | — |  | — | — |  |
| Yes |  | 1.63 | 0.98, 2.71 | 0.059 |  |  |  |  |  |  |  | 2.32 | 1.30, 4.15 | 0.004 | 1.48 | 0.96, 2.28 | 0.079 |
| *^1^* HR = Hazard Ratio, CI = Confidence Interval | | | | | | | | | | | | | | | | | |
